# The Fukushima nuclear accident and the pale grass blue butterfly: evaluating biological effects of long-term low-dose exposures

**DOI:** 10.1186/1471-2148-13-168

**Published:** 2013-08-12

**Authors:** Atsuki Hiyama, Chiyo Nohara, Wataru Taira, Seira Kinjo, Masaki Iwata, Joji M Otaki

**Affiliations:** 1 The BCPH Unit of Molecular Physiology, Department of Chemistry, Biology and Marine Science, Faculty of Science, University of the Ryukyus, 1 Senbaru, Nishihara, Okinawa 903-0213, Japan

**Keywords:** Abnormality rate, Artificial radionuclides, Colour pattern, Fukushima nuclear accident, Long-term low-dose radiation exposure, Pale grass blue butterfly, *Zizeeria maha*

## Abstract

**Background:**

On August 9th 2012, we published an original research article in *Scientific Reports*, concluding that artificial radionuclides released from the Fukushima Dai-ichi Nuclear Power Plant exerted genetically and physiologically adverse effects on the pale grass blue butterfly *Zizeeria maha* in the Fukushima area. Immediately following publication, many questions and comments were generated from all over the world. Here, we have clarified points made in the original paper and answered questions posed by the readers.

**Results:**

The following points were clarified. (1) There are many advantages to using the pale grass blue butterfly as an indicator species. (2) The forewings of the individuals collected in Fukushima were significantly smaller than in the northern and southern localities. (3) We observed growth retardation in the butterflies from the Fukushima area. (4) The aberrant colour patterns in the butterflies obtained in the Fukushima area were different from the colour patterns induced by temperature and sibling crosses but similar to those induced by external and internal exposures to the artificial radionuclides and by a chemical mutagen, suggesting that genetic mutations caused the aberrations. (5) This species of butterfly has been plentiful in Fukushima area for at least half a century. We here present specimens collected from Fukushima Prefecture before the accident. (6) Mutation accumulation was detected by the increase in the abnormality rates from May 2011 to September 2011. (7) The abnormal traits were heritable. (8) Our sampling localities were not affected by the tsunami. (9) We used a high enough number of samples to obtain statistically significant results. (10) The standard rearing method was followed, producing normal adults in the control groups. (11) The exposure experiments successfully reproduced the results of the field work. This species of butterfly is vulnerable to long-term low-dose internal and external exposures; however, insect cells are known to be resistant to short-term high-dose irradiation. This discrepancy is reconcilable based on the differences in the experimental conditions.

**Conclusions:**

We are just beginning to understand the biological effects of long-term low-dose exposures in animals. Further research is necessary to accurately assess the possible biological effects of the accident.

## Background

Because of the Great East Japan Earthquake, which occurred on March 11th 2011, the Fukushima Dai-ichi Nuclear Power Plant (NPP) exploded and released a massive amount of artificial radionuclides into the environment. Next to the Chernobyl accident in 1986, this was the second largest environmental pollution caused by a nuclear power plant accident in history. Although there have been several studies of the biological effects of the Chernobyl accident, the biological effects of long-term low-dose exposures are still under debate because, to the best of our knowledge, none of the studies continuously monitored the effects on ecosystems or on a particular organism from the very early stages post-accident. In addition, none of the studies reproduced the field-based results in the laboratory through genetic crosses and external and internal exposure experiments using an indicator species.

**Figure 1:**
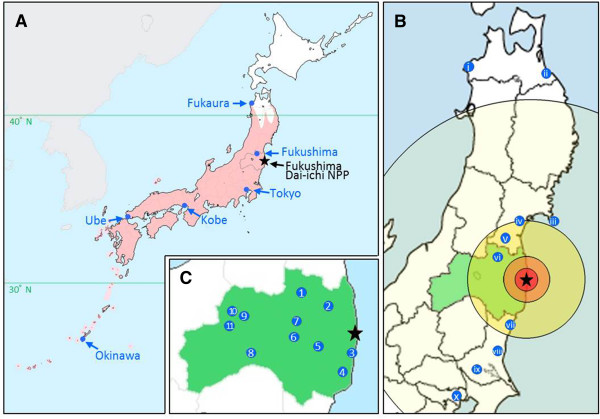
**Important localities that appear in this paper, and the latest distribution of the pale grass blue *Zizeeria maha*.** The Fukushima Dai-ichi NPP is indicated with black stars. **(A)** Map of Japan. The pink regions indicate the possible latest distribution of the pale grass blue. The northern range margins were estimated based on our own field work and Shirôzu (2006) [8], **(B)** Map of the Kanto-Tohoku district in Japan. The radii of the circles measure 20, 40, 100, and 300 km from the Fukushima Dai-ichi NPP. The Roman numerals i-x indicate the localities as follows: (i) Fukaura, (ii) Hachinohe, (iii) Kinkazan, (iv) Sendai, (v) Shiroishi, (vi) Fukushima, (vii) Takahagi, (viii) Mito, (ix) Tsukuba, and (x) Tokyo. **(C)** Map of Fukushima Prefecture (coloured in green). The Arabic numerals 1-11 indicate the localities from which the specimens were collected in May and September 2011 after the accident and from which specimens were collected before the accident as follows: (1) Fukushima, (2) Iitate, (3) Hirono, (4) Iwaki, (5) Ono, (6) Koriyama, (7) Motomiya, (8) Shimogo, (9) Aizu-Sakashita, (10) Takasato (Kitakata), and (11) Mishima.

Recognising this emergent need, we investigated the biological effects of the Fukushima accident at a very early stage of the contamination through genetic crosses and external and internal experiments using the common lycaenid butterfly, the pale grass blue *Zizeeria maha* (Kollar, 1844) (Lepidoptera, Lycaenidae), as an indicator species [1]. We have been studying *Z. maha* in the field and the laboratory for more than a decade [1-5], and we have established a rearing method that is specific to this species and can be used as the basis for physiological and genetic studies of this butterfly [2]. In May 2011 and September 2011, approximately two months and six months after the accident, respectively, we performed field sampling of this butterfly in and around Fukushima Prefecture (Figure 1). The crossing and rearing experiments were performed at the University of the Ryukyus, Okinawa, one of the regions in Japan least affected by the accident because it is located 1,763 km from the Fukushima Dai-ichi NPP. We performed external and internal exposure experiments and successfully reproduced the results of the field work using the *Z. maha* individuals that were obtained in Okinawa and had almost never been exposed to artificial radionuclides from the Fukushima Dai-ichi NPP.

Immediately after the publication of our research in *Scientific Reports* on August 9th 2012 [1], we received numerous questions and comments from people around the world. According to the publisher’s website metrics (http://www.nature.com/srep/2012/120809/srep00570/metrics), as of January 22nd 2013, the original paper [1] has received an exceptionally large number (276,139) of views. A number of the comments were encouraging, whereas others were discouraging, and a number of the comments were scientific, whereas others were emotional and/or political. On the one hand, we very much appreciated the honest scientific discussions that contributed to our deeper understanding of the scientific truth. On the other hand, we received quasi-scientific or pseudo-scientific discussions that could be interpreted as political. It is important to distinguish the political issues from the scientific facts. We were not able to address the non-scientific comments in this report, although our advice to the authors of these comments is to read the original and related papers carefully.

However, to avoid scientific confusion, we are obliged to answer the scientific questions and to systematically address the scientific comments. To our surprise, we now know that biological field sampling attempts from immediately after the Fukushima accident are scarce, not to mention scarcity of experimental demonstration of irradiation effects. In this report, we explained the important points raised by our readers. To facilitate the understanding of the spatial relationships among the sampling localities, we have included maps of Japan, the Kanto-Tohoku district, and Fukushima Prefecture (Figure 1).

The main findings of the original publication [1] can be summarised as follows:

(1) We observed morphological abnormalities in the field-caught adult butterflies with respect to the wing shape, wing colour pattern, appendages, antennae, palpi, compound eyes, thorax, and abdomen. The frequency of these abnormalities in the adults did not correlate with the ground radiation dose in the localities where the specimens were collected in May 2011. In contrast, in the specimens collected in September 2011, a high positive correlation between the abnormality rate of adults and the ground radiation dose was observed. Similar morphological abnormalities were reproduced experimentally in the individuals externally exposed to ^137^Cs and in the individuals internally exposed through feeding with the natural host plant collected from the contaminated areas.

(2) In the May samples, the forewings of the male individuals from Fukushima were smaller than from other localities. Specifically, we observed a forewing-size reduction that was dependent on the ground radiation dose. In addition, this possible radiation effect on the forewing size was reproduced in the external and internal exposure experiments.

(3) The abnormality rates of adults in the September field samples were higher than those in the May field samples. Similarly, the abnormality rates of adults in the September F_1_ samples were much higher compared with the May F_1_ samples.

(4) The offspring (F_1_ generation) of the field females (P generation) collected in May exhibited various morphological abnormalities. Among the abnormalities, the abnormality rate of the appendages demonstrated a high inverse correlation with the distance of the collection localities from the Fukushima Dai-ichi NPP.

(5) In the F_1_ generation from the May and September samples, the growth periods to pupation and to eclosion were longer in the samples collected from the localities closer to the Fukushima Dai-ichi NPP. That is, we observed growth retardation that was inversely dependent on the distance from the NPP.

(6) The F_2_ generation, generated from genetic crosses between the abnormal F_1_ individuals from the various localities and the normal F_1_ individuals from Tsukuba in the May samples, demonstrated the inheritance of the abnormalities. Many of the abnormalities in the F_2_ generation were identical or homologous to those in the F_1_ generation. In addition, some F_1_ individuals were infertile or sterile.

(7) In the experiment using ^137^Cs as the radiation source for the external exposure of the Okinawa individuals, we observed a dose-dependent decrease in the survival rate.

(8) In the internal exposure experiments in which the contaminated host plant collected from the Fukushima area was fed to the Okinawa individuals, we observed a decrease in the survival rates that was proportional to the ^137^Cs activity and to the ^134^Cs activity contained in the host plant.

Based on these results, we concluded that at least some of the morphological and physiological abnormalities detected in the field-caught individuals from the Fukushima area and their progeny originated from the physiological and genetic damage to somatic and germ-line cells caused by the artificial radionuclides from the Fukushima-Dai-ichi NPP. Among this series of experiments, the most important was the internal exposure experiment, which demonstrated that the contaminated host plant directly caused the death of larvae and pupae in a dose-dependent manner. Alternative reasons for the internal exposure results are rather difficult, although not impossible, to explain (see Point 11). This experimental setting likely reflects what happened in the *Z. maha* in the Fukushima area. As far as we could determine, none of the comments from around the world criticised the internal exposure experiments and thankfully, many comments were positive. To our knowledge, this study is the first to use this experimental procedure (rearing animals collected from a non-contaminated area and feeding them contaminated foods collected from contaminated areas).

## Methods

### Re-analyses of the previous data

The numerical data presented in Supplementary Information of the *Scientific Reports* paper [1] were here re-analyzed and expressed in graphs and other visual aids that were not shown in the previous paper. One-dimensional scatter gram, box plots, bar graphs, and scatter plots were newly made using R version 2.1.4.2 (The R Foundation for Statistical Computing, Vienna, Austria). Statistical analyses were performed using R and JSTAT (Yokohama, Japan).

### New data in this paper

For a more in-depth discussion, we have included new data that have not been published. Abnormality rates of external and internal exposure experiments including their control rates that were presented in the previous paper [1] were shown in this paper for the first time. Colour-pattern changes were induced by cold shock or by sibling crosses, according to the previous studies [2-5]. Mutation-induced changes of colour patterns and legs were obtained by feeding larvae an artificial diet containing ethyl methanesulfonate (EMS), a common mutagen. Detailed methods for this mutagenesis experiments will be published elsewhere. We obtained specimens that were collected before the accident (see below), which were kindly gifted from lepidopterists in Fukushima Prefecture, N. Nagata and I. Tsunoda. Temperature data were obtained from Japan Meteorological Agency via internet (http://www.data.jma.go.jp).

### Retrieval of comments and eleven critical points

As many commentators indicated, we admit that more profound discussions are necessary to interpret and understand the data correctly. We investigated as many of the scientifically relevant questions and comments from members of our own group, those posted on the web, and those sent to us directly via e-mail as possible and summarised them into the following eleven points: [Point 1] The suitability of the pale grass blue butterfly as an environmental indicator or a laboratory animal. [Point 2] The possibility of latitude-dependent forewing-size reduction. [Point 3] The significance of the growth retardation. [Point 4] The possibility that the colour-pattern aberrations were caused by temperatures. [Point 5] The possibility of locality-dependent variants or abnormalities. [Point 6] The implications of the abnormality rates and the possible accumulation of genetic mutations. [Point 7] Whether the abnormalities were because of genetic mutations. [Point 8] The relevance of the sampling localities and the possible effects of the tsunami and earthquake. [Point 9] The relevance of the sample size. [Point 10] The relevance of the rearing conditions. [Point 11] The importance of the exposure experiments and the possible discrepancy in the radiation sensitivity.

In addition, a small number of the critics noted that there was no comparison of the current population of this butterfly with the population before the accident. Fortunately, thanks to Japanese amateur lepidopterists, we have successfully retrieved several specimens from the previous populations collected from Fukushima Prefecture before the accident, which is discussed in Point 5.

We have discussed these eleven points based not only on the original data [1] but also on the relevant literature on insect physiology. We believe that solid discussions based on the current knowledge of insect physiology and on the natural history of *Z. maha* are necessary, which regrettably were ignored in many of the comments.

## Results and discussion

### [Point 1] The suitability of the pale grass blue as an environmental indicator or a laboratory animal

The commentators argued that the pale grass blue is not suitable as an indicator species, which may originate from the fact that this butterfly is not popular amongst biologists. We would like to note that most model organisms are not suitable as indicators in field studies. For example, it is difficult to collect local wild types of *Drosophila melanogaster* anymore because of the possibility of contamination with experimental mutant strains and because of the heavy human (and therefore fly) traffic associated with fruit export and import. Additionally, we would like to note that previous studies have used *Z. maha* as an indicator species [6,7]. To understand the suitability of *Z. maha* as an indicator species, we have briefly discussed the natural history of this species below.

The pale grass blue *Z. maha* is distributed throughout Japan including Honshu, Shikoku, Kyushu, and the Ryukyu Archipelago (Okinawa Prefecture and Kagoshima Prefecture) [8] (Figure 1). The northern range margins are located in Fukaura (facing the Japan Sea) [3,8,9], which was confirmed by our latest field work in September 2012, and in Hachinohe (facing the Pacific Ocean), which is based on personal communication and information from the Ikari Corporation website (2008) (http://www.ikari.jp/column/c4/c4_rep24_20081007.html). Both Fukaura and Hachinohe are located in Aomori Prefecture (Figure 1) and are located far north of the Fukushima Dai-ichi NPP, at 372 km and 346 km, respectively. As the Japanese name *Yamato-shijimi* (meaning the Japanese lycaenid) implies, this butterfly is one of the most prosperous species, especially in human habitats, in Japan, including the Fukushima area around the Fukushima Dai-ichi NPP [8,10-12].

**Figure 2:**
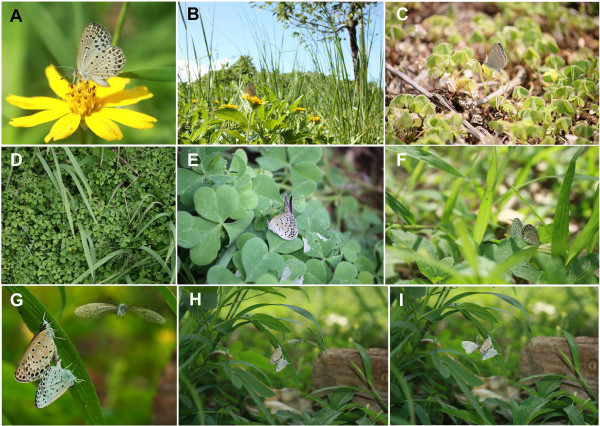
**Natural behaviour of the pale grass blue *Zizeeria maha *adults in the field. (A, B)** An adult visiting a flower that is close to the ground. Image taken by M. Iwata in the Nishihara Campus of the University of the Ryukyus. **(C)** An adult resting on a host plant, which is very close to ground. Image taken by W. Taira in Irabu-jima Island, Okinawa Prefecture. **(D)** The host plant community. Image taken by W. Taira in Hirono, Fukushima Prefecture. **(E)** Mating behaviour on the host plant. Image taken by W. Taira in Yatabe, Tsukuba, Ibaraki Prefecture. **(F-I)** Copulation. The large adult is female, resting on the grass close to the ground. In G-I, note the male adult flying close to the ground toward the copulating female. G is a high magnification of H. Images taken by M. Iwata in the Nishihara Campus of the University of the Ryukyus.

This butterfly is a specialist that thrives on the host plant *Oxalis corniculata* (Figure 2). The monophagous nature of the butterfly is important for its use as an indicator species because the life history of the butterfly is dependent on this plant, which simplifies the field work, laboratory work and interpretation of the data. The host plant is often found in rice and vegetable fields, grasslands and gardens; therefore, *Z. maha* is often found in man-made environments, including urban areas. Importantly, *Z. maha* is multi-voltine, completing its life cycle in one month. On the Honshu mainland, including the Fukushima area, the adults emerge mainly from late April to late October. During this six-month period, the generations repeat five to six times continuously. In Okinawa, the adults can be found throughout the year, which is an extra advantage of using this species in our laboratory located in Okinawa.

In Japan, *Z. maha* is most likely one of the weakest butterflies in terms of flying ability. The adult butterfly flies mostly at a height of less than 1 m, partly because its host plant is usually less than 10 cm high. Moreover, the adult life span is approximately one week (under our rearing conditions). Based on single-individual tracing field work of *Z. maha*, a single adult of this species disperses over only a limited distance [13], likely a few kilometers at most. Thus, the dispersion of *Z. maha* in a single generation is more limited than any other butterfly in Japan and accordingly, the dispersion can be ignored when interpreting the results of the field work. Practically no other butterfly species in Japan is easier to collect, which allows researchers to collect relatively large numbers of individuals that can be subjected to rigorous statistical analysis.

Because the host plant is small in height, *Z. maha* spends its entire life very close to the ground (Figure 2). Therefore, the butterfly is directly affected by the ground surface environment. This life history is advantageous for monitoring the biological effects of radionuclide contamination because the radioactive fallout accumulates on the ground. When the meltdown and explosion of the Fukushima Dai-ichi NPP occurred, the larvae were overwintering, sitting still on the ground surface on the host plant. Therefore, it is certain that the overwintering larvae were exposed externally to the artificial radiation from the very first day of the NPP explosion. In addition, the larvae ate the contaminated host plant before pupation, leading to simultaneous external and internal exposures.

**Figure 3:**
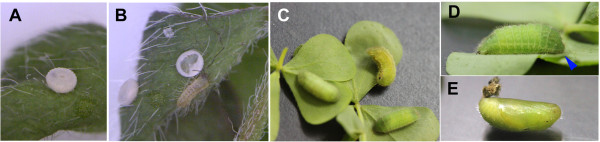
**Life stages of the pale grass blue *Z. maha *in the laboratory. (A)** Egg. **(B)** Hatched first-instar larva. **(C, D)** Late-instar larvae. The arrow in D indicates the head. **(E)** Pupa.

We have established a standard rearing method that can produce successive generations of this species (Figure 3) [2]. The establishment of a rearing method is required for laboratory animals. Once established, the rearing procedure is relatively simple, and under our standard conditions (25-27°C, 16L-8D), we obtained morphologically and behaviourally normal adults [2]. Because this butterfly is small, a large number of individuals can be reared in a small laboratory space.

**Figure 4:**
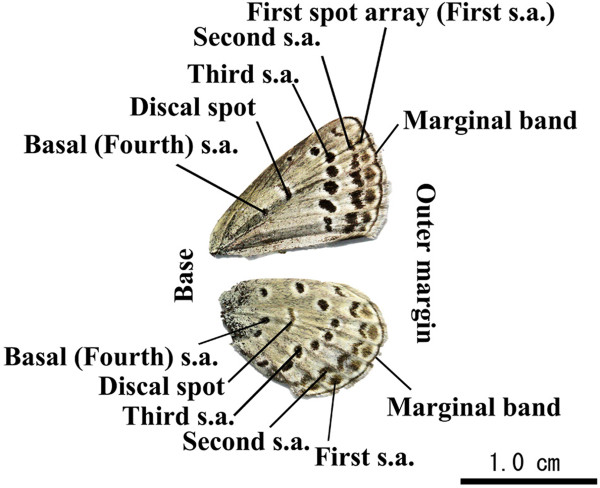
**Nomenclature of spots on *Z. maha *wings.** The wings shown are from a female individual (spring morph) caught in Tsukuba, Ibaraki Prefecture, Japan during the sampling in May 2011. Scale bar, 1.0 cm.

The colour pattern of *Z. maha* is relatively simple. The dorsal side is sexually dimorphic but the ventral side is not. On the ventral side, there are black spots arranged regularly in four arrays and a single discal spot against a light grey background with no additional colouration (Figure 4). Thus, it is easy to compare the colour patterns among individuals and to evaluate aberrations, if any. The normal colour pattern of *Z. maha* is stable, at least under the standard rearing conditions [2].

However, the colour pattern changes in response to environmental stress [3-5], and this sensitivity makes *Z. maha* a useful species for monitoring environmental change. Although the background abnormality rate of adults in *Z. maha* is approximately 12.2% on average, we considered that the abnormality rate of adults more than 15% for field-collected samples and 20% for laboratory-reared samples warranted an explanation (see Point 6). A number of the critics claimed that the abnormalities reported in our previous paper [1] are within the normal variation levels for this species and that the frequent abnormalities observed in this species, even without any radiation exposure, make *Z. maha* unsuitable for this type of research. We do not agree. What are needed are reasonable control groups and the use of standard rearing conditions in the laboratory to produce consistently healthy *Z. maha* individuals (see Point 10).

Based on these advantages, we believe that *Z. maha* is one of the best environmental indicator species in Japan. The species is especially suitable for radionuclide contamination experiments because of its close association with human habitats, monophagous nature, high collectability (high frequency of encounter in the field), relatively simple rearing method, small body size (suitable for mass production), clear-cut colour-pattern evaluation system, weak flying ability (small level of dispersion), living close to the ground, multi-voltine nature, and short generation time. As a laboratory animal, *Z. maha* is likely the first lycaenid butterfly system that allows researchers to work on hundreds or thousands of individuals, allowing statistical evaluations (see Point 9). Nonetheless, we have not dismissed the possibility that other organisms may be equally or more suitable as an indicator species.

### [Point 2] The possibility of latitude-dependent forewing-size reduction

Some commentators were unsure of our data on forewing-size reduction. We demonstrated that the forewings in the male individuals from Fukushima were smaller than in the males from Hirono, Takahagi, Tsukuba, and Tokyo [1]. These differences were statistically significant. Moreover, the forewing-size reduction correlated with the radiation dose. Importantly, we reproduced the forewing-size reduction in the external and internal exposure experiments (see Point 11). Therefore, we are confident in our radiation-induced forewing size reduction data for this species.

However, we are sorry that we might have generated confusion by our representation of the box plot (Figure 1c in the original paper [1]) in which Fukushima appears to be the northernmost city from which samples were obtained. This could be interpreted by the readers as an “edge effect”. In reality, the northernmost city from which we sampled was Shiroishi, but in the original box plot, we did not include the Shiroishi samples. One of the three respectable reviewers of the original *Scientific Reports* paper [1] (all three reviewed the manuscript quite deeply before publication; this communication paper was also reviewed by three reviewers) suggested not presenting the box plot or removing the Shiroishi data from the box plot because of the small sample size. Because this was a reasonable request based on current statistical conventions, we agreed with the reviewer and removed the Shiroishi data from the figure.

**Figure 5:**
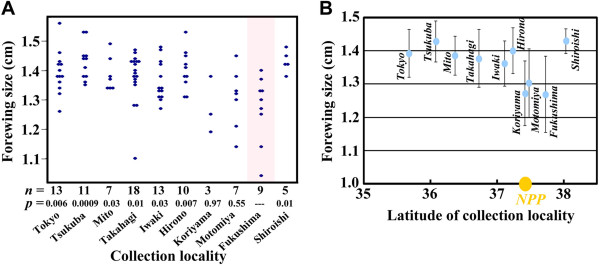
**Forewing-size differences among male individuals of the pale grass blue *Z. maha *caught in May 2011. (A)** One-dimensional scatter gram including the Shiroishi samples, which were not included in the box plot in the original paper [1]. The distribution in Fukushima is shaded in light pink. The *p*-values (after Holm correction) obtained by *t*-test (Fukushima versus the rest) were shown. We confirmed the equality of variance using the *F* test and the normality using the Shapiro-Wilk test and the Kolmogorov-Smirnov test. **(B)** A relationship between latitude of collection locality and forewing size. Each plot represents mean ± SD. Localities close to the nuclear power plant (NPP) showed smaller forewing size.

However, this does not mean that our Shiroishi data were invalid. In this report, we have included a one-dimensional scatter gram that includes the Shiroishi data (Figure 5). Figure 5 clearly indicates that the Fukushima (and Motomiya) samples were smaller than the northern and southern locality samples and excludes the possibility of an edge effect. In addition, the forewing-size reduction was shown to be dose-dependent, as demonstrated in Figure 1d in the original paper [1], which included the Shiroishi samples.

Some critics argue that the forewing-size reduction observed in the individuals collected from the Fukushima area was a result of high-latitude or low-temperature effects because the Fukushima area appears to be close to the northern range margin for this species. This statement contains two errors. First, the Fukushima area is not close to and is actually far from the northern range margin for this species. The northernmost range margin is Fukaura in Aomori Prefecture [3,8,9], which is located 370 km north of the Fukushima Dai-ichi NPP (Figure 1). Second, generally speaking, high latitude and low temperature are associated with a larger, not smaller, body size. If there were any size clines in natural populations because of a latitude or temperature effect, it would be a latitude-dependent size increase instead of a size reduction compared to the southern populations. In Japan, the smallest individuals are found in Okinawa Prefecture in the southernmost populations of *Z. maha*. The body size of the Okinawa population is so small that they are considered a unique subspecies. Consistent with this, in a given locality, the first-voltine population (spring morph) has the largest body size [8,11,14]. Our data, which now includes the Shiroishi samples, did not demonstrate a simple cline, as seen in correlation coefficient *r* = -0.43 and *p* = 0.21 between latitude and forewing size (Figure 5). Instead, our data demonstrated an abrupt and statistically significant size decrease in Fukushima (together with Motomiya and Koriyama; these three localities are closely located together), compared with both the northern and southern localities. We also note that size variation represented by error bars in Figure 5 was larger in Fukushima (and its close localities, Motomiya and Koriyama) than other localities. Indeed, there were exceptionally small individuals in Fukushima and its close localities. These facts implicitly indicate that a possible causal factor for the size reduction is not a general factor, such as latitude and climate, that is imposed equally on all individuals in a given locality, but it is a relatively specific factor, such as large-scale pollution, that is imposed differently on different individuals due to variation in genetic composition (inherent resistance or sensitivity) and habitat microenvironment (where larvae and pupae lived at the time of pollution).

Generally, the body size of animals, including insects, is larger under low-temperature conditions (known as temperature-size rule), and this rule applies to more than 80% of ectotherms including *Drosophila*[15-18]. Low-temperature rearing increases the body size of *Drosophila*[19-22]. Regrettably, some critics cited the case of *D. subobscura*, which demonstrates rapid size adaptation in North America [23,24], to argue that our results (i.e., small forewing size in the Fukushima samples) were simply the natural size for the species because of the high latitude and cold temperatures. Contrary to their expectations, the *D. subobscura* case actually supports our finding because these studies confirm that larger (not smaller) size is found in high-latitude cold regions [23,24].

To be sure, the temperature-size rule does not apply to some groups of insects. At least in univoltine or bivoltine orthopteran species (i.e., grasshoppers and crickets), body size is dependent on how many generations per year can be achieved, suggesting that body size is adaptation to season length [25,26]. The pale grass blue is a multivoltine lepidopteran insect, suggesting that it is not an exception of the temperature-size rule.

We are certain that the forewing-size reduction we observed in Fukushima [1] reflected the body size reduction because their smallness was easily visualised during rearing, although this has not been demonstrated quantitatively. The use of an artificial diet that does not contain the complete nutrients that this butterfly needs appears to reduce the body size and thus the forewing size in *Z. maha*[2]. Starvation at the larval stage can also produce small body size and small forewing size [2]. Importantly, we reproduced this size reduction in the external and internal exposure experiments [1], although the mechanisms remain unclear. The irradiation effects in the midgut cause nutrient deficiency in adult weevils [27]. A similar effect could contribute to the wing-size reduction in the irradiated *Z. maha* individuals.

The forewing-size reduction was a physiological effect (that could involve somatic mutations), not a genetic effect (that involves germ-line mutations), because the May samples were only exposed to the radiation during the larval and pupal stages. The May samples were the first-voltine individuals (spring morph) that were overwintering as mid- or last-instar larvae at the time of the accident. Therefore, these individuals were exposed to the radiation for approximately two months (March and April). In addition, the major radionuclide species at the time was iodine, which is much stronger in activity than caesium [28]. These conditions might have caused the size reduction of the first-voltine individuals. In contrast, we did not observe a similar size reduction in the September samples. The reason is not apparent, considering that we reproduced similar size reduction in external and internal experiments. This may be because of sample heterogeneity or natural selection for larger individuals in the wild. Experimental use of radioactive iodine in addition to caesium could resolve this issue, but ironically, such an experiment would be difficult to perform under tight Japanese regulations on radioactive materials.

To conclusively determine the possibility of latitude-dependent or pollution-dependent size changes, we are now analysing many *Z. maha* individuals collected from various regions throughout Japan (with the cooperation of amateur lepidopterists).

### [Point 3] The significance of growth retardation

**Figure 6:**
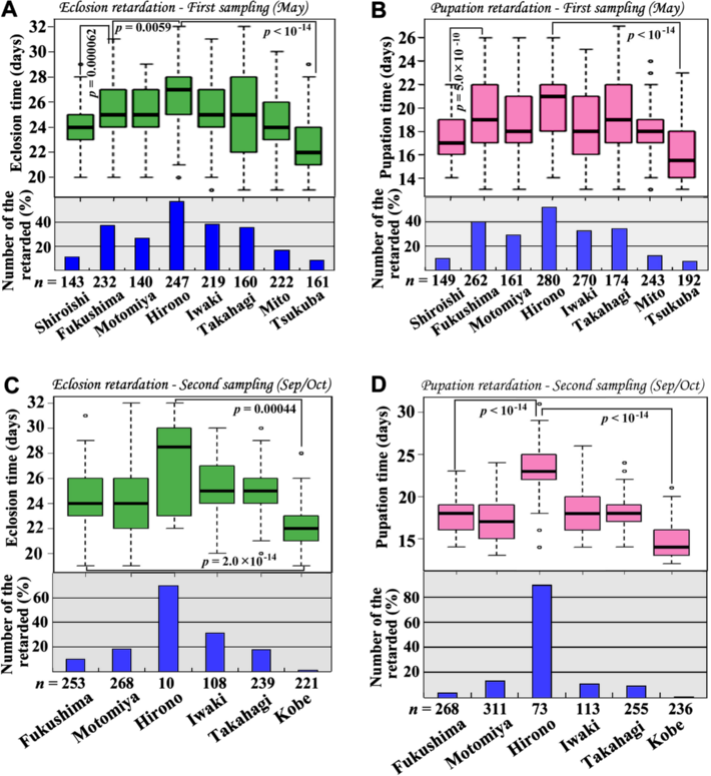
**Locality-dependent growth retardation of the pale grass blue *Z. maha*.** To obtain individual data on the eclosion and pupation times, pupated and eclosed individuals were recorded every day. The days of pupation and eclosion were counted starting from the very first day of the setting egg collection for each locality group. These data were used to construct the box plots. Statistically significant differences were obtained using the Kruskal-Wallis test in all four cases (*p* < 2.2 × 10^-16^) (A-D). The Steel-Dwass test was performed as a post-hoc test. All combinations with Tsukuba or Kobe were highly significant. Several combinations with significant *p*-values are shown. In addition, we constructed bar graphs in which a growth-retarded individual was defined as one that pupated 21 days or later after egg collection and one that eclosed 27 days or later after egg collection. These time points were set as dates of 99% pupae eclosed or 99% larvae pupated in the Kobe samples. The numbers of eclosed individuals are smaller than the number of pupated individuals because of pupal death. **(A, B)** Eclosion time (A) and pupation time (B) (box plots), and the number of the growth-retarded individuals toward eclosion (A) and pupation (B) (bar graphs) in the May samples. Note that the most retarded locality group was Hirono, the locality closest to the NPP. **(C, D)** Eclosion time (C) and pupation time (D) (box plots), and the number of the growth-retarded individuals toward eclosion (C) and pupation (D) (bar graphs) in the September/October samples.

We observed slow growth rates in the samples from many localities (Figure 6 in this paper and Figure 2 in the original paper [1]). This slow growth rate, together with the forewing-size reduction (and therefore the body-size reduction) discussed in Point 2, suggested that the larvae took longer to become an adult even though the final adult was smaller. In general, the growth period is longer in colder environments, resulting in a larger body size. However, we observed the opposite in our samples, which can be considered an abnormal physiological trait.

**Figure 7:**
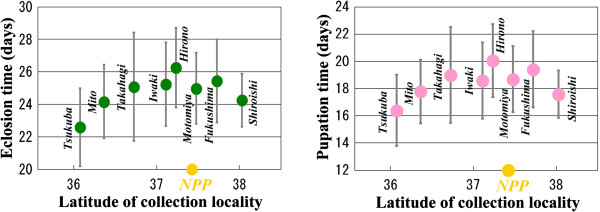
**Relationship between latitude and eclosion time or pupation time.** Each spot indicates mean ± SD. Latitude of the Fukushima Dai-ichi Nuclear Power Plant is indicated by a yellow dot (NPP). **(A)** Eclosion time. **(B)** Pupation time.

The observed growth retardation was inversely correlated with the distance from the NPP [1]. This phenomenon is most apparent in the eclosion retardation data from the May samples (Figure 6A). We observed more growth retardation in the Fukushima May samples than in the Shiroishi May samples, which excluded the possibility of an edge effect (Figure 6A, B), as discussed in Point 2. For the eclosion and pupation data in both the May (Figure 6A, B) and September/October samples (Figure 6C, D), the most affected population was from Hirono, approximately 20 km from the NPP. We further examined a possible relationship between latitude and eclosion time (and pupation time). Growth retardation peaked around the latitude of the Nuclear Power Plant (Figure 7). These results support growth retardation and also wing-size reduction effected by the accident, excluding the possibility of latitude, temperature, and edge effects.

### [Point 4] The possibility that the colour-pattern aberrations were caused by temperatures

**Figure 8:**
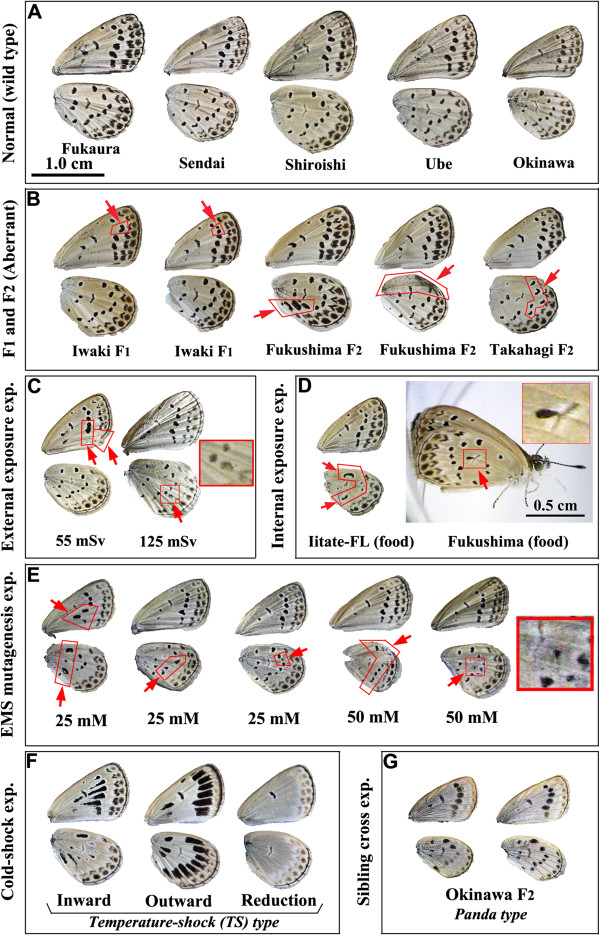
**Variations in wing colour patterns in the pale grass blue *Z. maha*.** The scale bar applies to all the panels in this figure, except for the right panel in D. The aberrant colour-pattern elements are boxed in B-E and are indicated with arrows. **(A)** Field-collected normal colour patterns. **(B)** F_1_ and F_2_ aberrant individuals from the May 2011 samples. The arrows and boxed areas indicate abnormalities. **(C)** Aberrations in Okinawa individuals obtained in the external exposure experiment. **(D)** Aberrations in Okinawa individuals fed with the host plant collected from the Iitate-flatland (FL) and Fukushima. **(E)** Aberrations induced by 25 mM or 50 mM EMS. Details of the experimental conditions and colour-pattern analysis will be published elsewhere. **(F)** Temperature-shock (TS) type. Inward TS type (left): Okinawa F_1_, cold-shocked at 4°C for 10 days. Outward TS type (middle): Okinawa F_5_ (selection line [3]), cold-shocked at −2°C for 3 days. Reduction TS type (right): Okinawa F_1_, cold-shocked at −2°C for 3 days. **(G)** Panda type: Okinawa F_2_ from a sibling cross.

Before discussing the colour-pattern aberrations, it is necessary to define the normal spot arrangement in this species (Figure 8A). Fortunately, the normal colour pattern is simple enough for comparison purposes but is complex enough that the detection of aberrations is obvious. The normal colour pattern of *Z. maha* from the wing margin to the wing base is composed of the first spot array, second spot array, third spot array, discal spot, and fourth (basal) spot array (Figures 4 and 8A). When the larvae experience low temperatures, a number of the spots may be reduced in size or may even disappear together with a darker background colouration [8,11,14]. However, the normal colour pattern is fundamentally stable under normal natural conditions throughout the year.

The pale grass blue *Z. maha* may be one of the species most frequently reported in lepidopterist journals for aberrant colour patterns. We previously reviewed two major Japanese monthly lepidopterology journals (*Chouken Field* and *Butterflies*) and found 16 reports of aberrant colour patterns in this species from 1987 to 2000 from many different localities [3], representing approximately one case per year. This high frequency of reports is likely proportional to the highest frequency of encountering this species in the field among butterfly species. Interestingly, all of the reports were cases of temperature-shock (TS)-type modifications (see Figure 8F).

The TS phenotype that was field-collected or induced in the laboratory by the application of temperature shock are classified into three types: inward, outward, and reduction types [3-5] (Figure 8F). In the inward TS type, the third and fourth spot arrays are elongated toward the discal spot. In the outward TS type, the spot arrays are elongated away from the discal spot, and in the reduction TS type, the spot arrays are reduced in size or are absent.

The TS-type individuals that are occasionally caught in the field are sporadic. However, the northern range margin population of this butterfly exceptionally exhibited a population-level emergence (more than 15%) of the TS-type individuals in Fukaura (Aomori Prefecture) from 2000 to 2005, especially in summer [3-5]. This TS phenotype is genetically fixed and thus expressed in summer without any temperature shocks. Interestingly, this outbreak was accompanied by the northern expansion of the distribution range, possibly in response to temperature warming. The Fukaura case can be considered a real-time field case of the evolution of colour patterns [3-5]. Notably, most modified individuals were normal with the exception of the colour pattern. It is important to emphasise that this outbreak was found only in Fukaura from 2000 to 2005. A similar case has not been reported in the northern range margin Hachinohe, and a similar outbreak has not been reported elsewhere. There is no evidence to suggest that *Z. maha* in the Tohoku district is generally abnormal or generally demonstrates a high incidence of abnormalities.

It is also important to stress that the TS-type colour-pattern modifications in the butterflies are expressed as plastic traits and not as pathological characteristics [3-5,29-34]. This is an important consideration when distinguishing the Fukushima case from the TS-type modifications. The Fukushima case is pathological, and the TS phenotype (including the Fukaura case) is not. The TS phenotype can be explained by phenotypic plasticity but the Fukushima case cannot. It is also important to recognise that the various and unpredictable abnormal patterns obtained in the Fukushima individuals (Figure 8B) were similar to those observed in the external and internal exposure experiments (Figure 8C, D).

Furthermore, an important feature of the Fukushima aberrations is their diversity (Figure 8B). This diversity is expected if the radiation caused random mutations. Indeed, the diverse Fukushima aberrations were similar to the mutants produced by the random mutagen, ethyl methanesulfonate (EMS) (unpublished data) (Figure 8E). EMS is a strong mutagen that causes random point mutations by directly damaging the DNA [35,36]. In the individuals collected from Fukushima, in the radiation-exposed individuals (both external and internal), and in the EMS-treated individuals, we observed similar diverse colour-pattern aberrations including spot fusion, spot addition, and spot disorganisation. We also observed wing-vein malformation. These diverse colour-pattern aberrations suggested that the damage was introduced at the DNA level. Moreover, the colour-pattern aberrations in the Fukushima individuals were inherited [1], suggesting that the aberrations were likely caused by genetic mutations.

Additionally, we previously showed that sibling crosses produced a unique pattern, which we termed the panda type [2] (Figure 8G). This phenotype is considered an indication of genetic deterioration. The panda type is easily distinguishable from other wing aberrations, and none of the field-collected and laboratory-reared samples from the original research [1] was similar to the panda type.

Taken together, the possibility that the colour-pattern aberrations we described in the original paper [1] were caused by temperatures is minimal and is not realistic. However, there is a possibility that a small number of the aberrations were actually the TS phenotype (despite the small degrees of modifications) that was caused by genetic mutations (not by temperatures) due to the artificial radionuclides from the NPP. We have discussed this possibility along with the genetic mutations in Point 7.

### [Point 5] The possibility of locality-dependent variants or abnormalities

The critics argued that the abnormality differences or growth differences among the different localities might have existed even before the accident. However, considering the very high abnormality rates detected in some localities, this possibility is unrealistic. As discussed in Point 4, the wing colour-pattern aberrations of *Z. maha* reported in the two major Japanese lepidopterology journals from 1987 to 2000 were of the TS phenotype [3]. The lepidopterology journals contained no reports of spot fusion, spot addition, spot disorganisation, or other abnormalities that were similar to the aberrations reported in our original paper [1].

In addition, other than the wing colour-pattern aberrations, most of the abnormalities detected in the F_1_ and F_2_ generations are likely fatal in the wild; the abnormal individuals could not reproduce offspring because of low fitness. It is difficult to believe that these high abnormality rates have been maintained in these populations for many years. The F_1_ generation also demonstrated low fertility (see Supplementary Table 5 in the original paper [1]), which means that the high abnormality and infertility rates detected in some localities would be impossible to maintain for long periods.

As expected, it has been demonstrated that *Z. maha* was the most successful species in the Fukushima area in 2004 and 2005 [11]. Indeed, in 1936, *Z. maha* was found in Kinkazan (Miyagi Prefecture), located 108 km north of the NPP [37,38], and *Z. maha* has been common in Fukushima Prefecture for at least half a century [10]. Therefore, the most likely explanation is that the high abnormality and infertility rates developed recently, probably right after the nuclear accident, and the *Z. maha* populations in the Fukushima area are now deteriorating at least at the time of the publication of the original paper [1].

**Figure 9:**
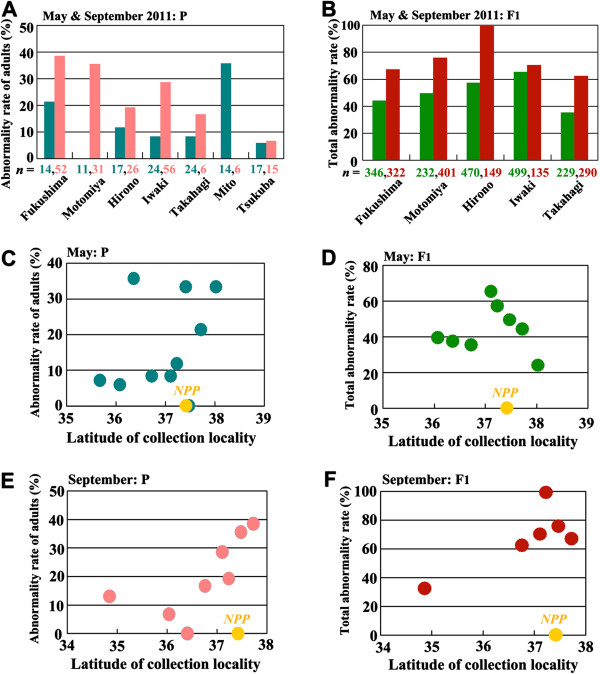
**Abnormality rates in different localities.** The original data for these graphs were presented in Supplementary Information of the original paper [1]. **(A)** Abnormality rates of adult samples field-collected in May (blue-green) and September (pink) 2011 in seven localities. With the exception of Mito (considered an outlier), the abnormality rates increased from May 2011 to September 2011. **(B)** Total abnormality rates of the F_1_ generation from the May and September field-caught parents in five localities. In all localities, the abnormal rates increased from May 2011 to September 2011. **(C)** Scatter plot to determine a possible relationship between the latitude of the collection locality and the abnormality rate in the adults in the field-caught May samples (the P generation) (*r* = 0.34, df = 8, *p* = 0.34). **(D)** Scatter plot to determine a possible relationship between the latitude of the collection locality and the total abnormality rate of the F_1_ generation from the May samples (*r* = −0.027, df = 6, *p* = 0.95). **(E)** Similar plot for the field-caught September samples (the P generation) (*r* = 0.70, df = 6, *p* =0.056). **(F)** Similar plot for the F_1_ generation from the September samples (*r* = 0.79, df = 4, *p* = 0.064).

According to our field sampling, the abnormality rates of adults in the seven localities varied [1] (Figure 9A). In all the localities, with the exception of Mito (considered an outlier), the abnormality rates of adults doubled or even tripled from May 2011 to September 2011. The highest rate in September was found in Fukushima (38.5%), suggesting a sudden environmental change. Additionally, in the September samples, we observed high correlation between ground radiation dose and abnormality rate (see Figure 4b in the original paper [1]), which was not observed in the May samples.

In addition to the abnormality rates of adults (Figure 9A), we compared the “total” abnormality rate (“total” means the inclusion of all abnormalities in the adults and the deaths of the larvae, prepupae, and pupae) in the May and September F_1_ generation samples (Figure 9B). In all five localities, the September samples exhibited larger abnormality rates than the May samples. We did not observe a latitude effect in the abnormalities in the P generation (*r* = 0.28, df = 8, *p* = 0.44) and the F_1_ generation (*r* = −0.0054, df = 6, *p* = 0.99) from the May samples (Figure 9C, D), although we admit that relatively small number of sampling localities might require a caution. If anything, the localities closer to the latitude of the Fukushima Dai-ichi NPP showed higher abnormality rates. Furthermore, similar plots of the September samples indicated peaks near the latitude of the NPP (Figure 9E, F). Although they showed relatively high correlation coefficients (*r* = 0.70, df = 6, *p* = 0.06 for the P generation; *r* = 0.79, df = 4, *p* = 0.06 for the F_1_ generation), this is because of scarcity of localities in the northern areas. Because *Z. maha* is well adapted to the Fukushima area [10,12] (see Points 1, 2, and 4), it is unreasonable to suggest latitude-dependent abnormalities in this species. The abnormality rates of adults are discussed further in Point 6.

The critics argued that because there is no record of populations from before the accident, our study is not valid. This comment is too extreme to consider rational because it suggests that any evolutionary studies that attempt to recapitulate the past based on data from existing organisms are invalid. Non-biologists should note that evolution has been a central theme in biology since Darwin. However, we agree that direct records from past populations would be informative.

**Figure 10:**
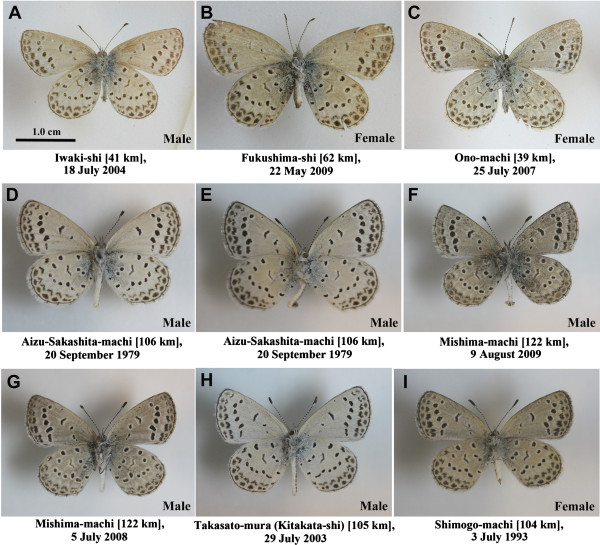
**Specimens of *Z. maha *collected in Fukushima Prefecture before the nuclear accident.** Distance of the collection locality from the Fukushima Dai-ichi NPP is shown. **(A-C)** Kind gift from N. Nagata. **(D-I)** Kind gift from I. Tsunoda.

It is likely that the largest number of amateur lepidopterists in the world reside in Japan. Thanks to these respected Japanese lepidopterists, we were fortunate to obtain nine specimens of *Z. maha* collected from different localities in Fukushima Prefecture between the years 1979-2009 (before the accident) (Figure 10; see Figure 1C for the collection localities). All of the specimens exhibited the normal colour pattern. The evaluation of abnormalities in other parts of these relatively old specimens was not possible because of physical damage to the appendages and other body parts. Nonetheless, the fact that all nine specimens exhibited a normal colour pattern suggests that in the past, the *Z. maha* in Fukushima Prefecture was normal. We note that this result is similar to a report in which no case of aberrant feathers was detected in the museum samples collected before the 1986 accident from the Chernobyl area [39].

### [Point 6] The implications of the abnormality rate and the possible accumulation of genetic mutations

**Figure 11:**
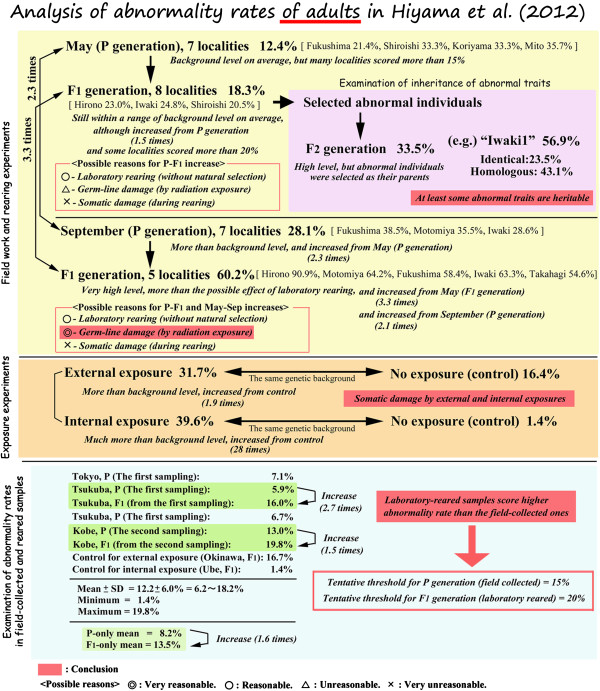
**Analysis of abnormality rates of adults.** Experimental results are in bold. Interpretations are in small italics. Conclusions are highlighted in red. The data were published in Hiyama et al. (2012) [1] except for the percentages of the controls for the external and internal exposure experiments.

It is important to understand the dynamic changes in the “abnormality rates of adults” among the different populations described in the original paper [1]. The critics frequently argued that our data were unusable because the background abnormality rate was too high to detect the influence of the radiation. We are sorry that we did not directly discuss the abnormality background levels in the original paper [1], although we did specify the abnormality rates in the Supplementary Information of the original paper [1]. Many readers misunderstood the abnormality rates, failing to differentiate the different rates, such as in the adult, larval, pupal and all stages. To clarify this issue, we have summarised the abnormality rates of adults in Figure 11. Note that in this figure, the abnormality rate of adults is discussed and not of the other stages or the total abnormality rates. Additionally, the rearing experiments were performed in an environment not contaminated with artificial radionuclides (i.e., in Okinawa). Therefore, the offspring were not exposed to artificial radiation, and the abnormalities beyond the background level may be attributed to the inheritance of the radiation effects from the parents.

At the bottom of Figure 11, we collectively presented the data for the normal populations that were not affected by the artificial radionuclides. The background rates of the P and F_1_ generations varied from 1.4% to 19.8% with a mean value of 12.2%. The background rates for the P generations varied from 5.9% to 13.0% with a mean value of 8.2%, whereas the F_1_ generation background rate varied from 1.4% to 19.8% with a mean value of 13.5%. In general, the laboratory-reared adults scored higher abnormality rates of adults than the field-collected ones. This is understandable because when reared in a laboratory, the larvae avoid predators and other selection pressures. Hence, even genetically unhealthy individuals can survive to adulthood, contributing to the increase in the abnormality rate of adults. In contrast, in the field, only relatively healthy adults can be collected.

For convenience, it is safe to set the tentative threshold for the P generation at 15% and for the F_1_ generation at 20%. Therefore, if the abnormality rate of adults is less than 15% in the P generation and less than 20% in F_1_ generation, this is considered background. However, if the abnormality rate of adults is more than 15% in the P generation or more than 20% in the F_1_ generation, this cannot be explained by background, and additional causes may be sought. A caution would be that even if the average rates are below the threshold levels, it does not mean that there is no effect of irradiation. In a specific locality or in a specific individual, there may be biologically significant effects, for example, even in the May samples (see below).

We will now address our field work and the rearing experiments in terms of the abnormality rates of adults. In May, we obtained an average abnormality rate of 12.4% in the P generation and 18.3% in the F_1_ generation. Both rates are within the range of background levels, and we concluded that this 1.5-times increase from the P to the F_1_ generation was a background increase because of the laboratory rearing discussed above. However, it should be noted that the abnormality rates at the independent localities tended to score more than 20%. In September, we obtained an average abnormality rate of 28.1% in the P generation and 60.2% in the F_1_ generation. Both values were more than the background threshold levels and were much higher than the May values.

To statistically test the difference between the May and September results, we first hypothesized that the abnormality rates of the May samples represent spontaneous level that is observed all the time in any *Z. maha* populations (that is, they are considered to be population mean values) and that their abnormality rates of adults in the P and F_1_ generations follow a binominal distribution. Because a binominal distribution can be approximated by a normal distribution when the number of samples are reasonably large, we were able to obtain expected mean (= *n* × *p*) and standard deviation (from a square root of variance; variance = *n* × *p* × (1 − *p*)) as 15 ± 3.62 (mean ± SD) for the May P samples and 207 ± 12.87 for the May F_1_ samples. When the September P and F_1_ samples were tested against the May P and F_1_ distributions, respectively, they were significantly different (*p* < 0.0001 for both cases; one-sample *t*-test). Similar results were obtained using *χ*^2^ test (*p* < 0.0018 for the P generation and *p* < 0.0001 for the F_1_ generation after Yates correction). Therefore, the September data needs an explanation beyond the spontaneous abnormalities and the laboratory-rearing effect. The cumulative germ-line damage by radiation exposure in the field is one of the most reasonable explanations for the increased abnormality rates observed in the September samples.

Among the localities examined, Motomiya in May is noteworthy. As indicated in the original paper, the adult collected from Motomiya did not exhibit abnormality at all. In other words, the abnormality rate of the Motomiya adults in the P generation was 0%. However, their F_1_ offspring exhibited a relatively high abnormality rate of adults (18.3%). Although this value was still below the tentative threshold for background (20%), the increase from 0% to 18%, which was the largest increase among the eight localities (see Supplementary Tables 2 and 4 in the original paper [1]), may be important.

To examine if the abnormal traits were heritable, we obtained the F_2_ generation from the May samples and estimated the abnormality rate of adults as 33.5% on average (Figure 11). Because we selected abnormal F_1_ individuals as the parents of the F_2_ generation, this high rate cannot be compared with other generations. This experiment was performed to demonstrate whether the abnormal traits were heritable. For example, in the strain named “Iwaki1”, 23.5% of the F_2_ progeny exhibited abnormal traits that were identical to the F_1_ parent, and 43.1% of the F_2_ progeny exhibited abnormal traits that were homologous to the F_1_ parents. These relatively high percentages suggest that at least some of the abnormal traits were heritable.

The gradual accumulation of mutations in the contaminated area occurs because of the continuous exposure of germ-line cells to radiation from generation to generation, which was suggested in the field samples by the increase in the abnormality rates of adults from May (12.4%) to September (28.1%). The generations from March 2011 to September 2011 in the wild were exposed to radiation; therefore, the genetic mutations accumulated in the germ-line genomes. In contrast, our laboratory is located in Okinawa, which was not affected by the accident; therefore, our laboratory-reared generations were not expected to show accumulation of genetic mutations.

It is worthwhile to note that the effects of radiation can be divided into two categories, the effects on somatic cells and on germ-line cells. The former effect could cause physiological disease in an irradiated individual, whereas the latter effect could cause genetic diseases in the progeny. The abnormal traits in the radiation-exposed generation (the P generation) do not directly contribute to abnormalities in their progeny because the pathological traits originate from somatic cells. In contrast, when germ-line cells are damaged, even if radiation-exposed individuals do not demonstrate pathological traits, their progeny could exhibit abnormal traits, because the somatic cells produced from the sperm and oocytes after fertilisation contained mutations and expressed pathological traits. Accordingly, we observed many abnormal traits in the F_1_ and F_2_ generations that were not observed in the P generation. The abnormalities in the F_1_ and F_2_ generations were more severe than in the P generation and were more numerous than in the laboratory-reared normal population.

It is reasonable to believe that in *Z. maha*, as in other insects, germ-line cells are more sensitive to radiation exposure than somatic cells. At the cellular level, the effects of irradiation are known as the Bergonié-Tribondeau rule in which the effects are proportional to the cell division frequency and the expected division number and are inversely proportional to the degrees of differentiation [40-44]. In lepidopteran insects, it is known that sperm become mature during the last instar and that oocytes become mature immediately before fertilisation [45]. At the time of the accident, the sperm were at least at the maturation stage. Because the germ-line cells are undifferentiated, they are more sensitive to radiation exposure than somatic cells.

### [Point 7] Whether the abnormalities were because of genetic mutations

Although we discussed the possible accumulation of genetic mutations in Point 6, we have only indirect evidence at this point that the abnormal traits we observed in the Fukushima-collected individuals originated from the mutation of genes [1]. In our experiment, at least some of the abnormal morphological traits found in the F_1_ generation were inherited by the F_2_ generation, indicating that these abnormal traits have genetic origins (see Point 6). The increase in the abnormality rate of adults from May to September also supports this interpretation. The germ-line damage in the first-voltine individuals caught in May 2011 is a likely possibility, as discussed above.

Importantly, the abnormal traits observed in the F_1_ and F_2_ individuals were similar to those observed in the EMS-induced mutagenesis experiment (unpublished data) (see Point 4, Figure 8). EMS is a widely used mutagen that causes random point mutations, supporting the notion that the abnormal traits observed in the field-collected individuals from the Fukushima area originated from genetic mutations.

Consistent with these results, we observed abnormal individuals with phenotypes similar to *Bar* and *Distal-less* mutants. A small number of the critics argued that many of the observed abnormal traits were biased to the legs, antenna, and wings; therefore, these non-random traits were unlikely to originate from random genetic mutations. We disagree. Phenotypic bias in a random mutagenesis experiment is common, and studies cannot obtain random abnormal traits. This bias arises for many reasons, including the limited ability to examine the morphology and nature of the mutated genes (e.g., expressivity, chromosomal locations, and physiological function). We have never claimed that the *Distal-less* gene is damaged more frequently than other genes. Instead, we claimed that when this gene and its related genes are mutated through a random mutation process, these mutations might be conspicuous as abnormal traits.

We admit that we observed spot elongation that was similar to the TS phenotype in many of the progeny from the Iwaki and Motomiya samples; however, these individuals were not comparable to the Fukaura individuals in the degrees of the modification. Because this spot-elongation aberration was inherited in our F_2_ generation experiment, it is likely that this aberrant phenotype was caused by genetic mutations. This argument is also supported by the female-biased inheritance of this trait; the TS phenotype is more frequently observed in females [3]. It is worthwhile stressing that the TS phenotype induced by temperature is considered a phenocopy of a genetic mutant, which has been indicated by esteemed scientists, such as Goldschmidt, Waddington, Shapiro, and Nijhout [46]; therefore, there are a small number of genes that cause the TS phenotype when mutated. It is reasonable that we detected a small number of aberrant individuals similar to the TS phenotype, caused by irradiation-induced random mutagenesis, in the Iwaki and Motomiya samples. We admit that there is a possibility that cold-shock-induced phenotypes are genetically fixed and are inherited in the Iwaki and Motomiya samples by a mechanism that does not involve the artificial radionuclides from the NPP. Even so, this possibility is applicable only to the TS phenotype and not the other aberrant traits that were inherited in the F_2_ generation.

**Figure 12:**
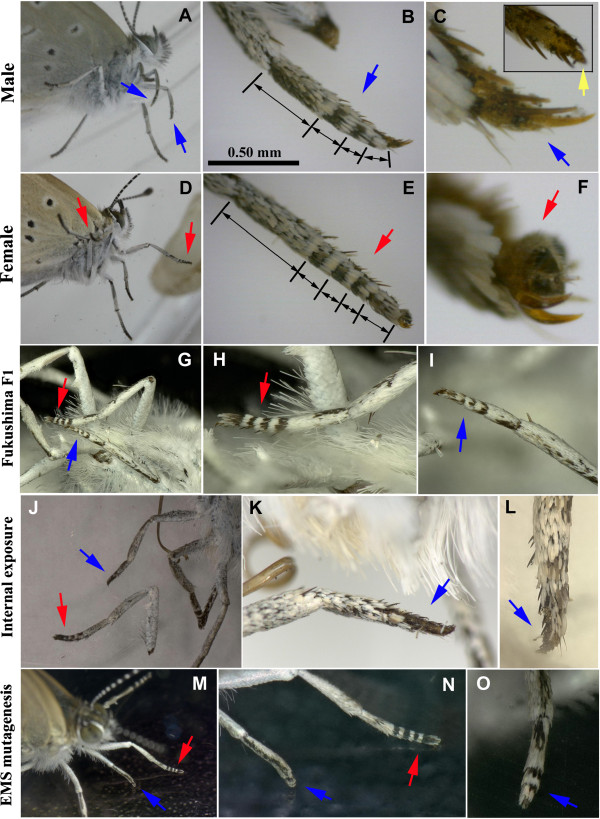
**Normal and gynandromorphic forelegs in *Z. maha*.** Blue arrows indicate male foreleg tips, whereas red arrows indicate female foreleg tips. **(A-C)** Male foreleg tarsus, containing four segments. Scale bar in B indicates 0.50 mm, which also applies in E. High magnification at the tip of the leg is shown in C. Inset shows the tip of the leg but from a different angle, revealing a possible remnant-like tarsomere, a fifth segment (yellow arrow). **(D-F)** Female foreleg tarsus, containing five segments. **(G-I)** An F_1_ individual from Fukushima having one male foreleg and one female foreleg. **(J-L)** An individual obtained in the internal exposure experiment having one male foreleg and one female foreleg. **(M-O)** An individual obtained by EMS mutagenesis having one male foreleg and one female foreleg.

In an area affected by the Chernobyl accident, an outbreak of gynandromorphic butterflies exhibiting both male and female wing colouration has been reported [47]. We did not observe wing gynandromorphs in our field work or experiments. However, we obtained a small number of gynandromorphs in which one of the forelegs was morphologically male and the other female in the Fukushima F_1_ generation, the EMS-mutagenesis experiment, and the internal exposure experiment (Figure 12). Note that *Z. maha* can be sexed easily using the structure of the forelegs. The gynandromorphs we obtained suggested genetic changes in the Fukushima individuals, the EMS-treated individuals, and internally exposed individuals, although we have not demonstrated the heritability of this trait and have no data on the site of the mutation at this point.

We do not exclude the possibility that a small number of the abnormal traits observed in the F_1_ and F_2_ generations of *Z. maha*[1] may correspond to epigenetic changes [48-52]. Among low-dose radiation effects, genomic instability can be passed on to the progeny by epigenetic factors, and DNA aberrations, chromosomal aberrations and other aberrations have been observed after several generations from irradiated parents [53-56]. Genomic instability is observed at the level of the cell and the organism [57-65]. A small number of the abnormal traits observed in the F_1_ and F_2_ generations of *Z. maha* in our study [1] may correspond to similar epigenetic changes; however, to determine this, it will be necessary to investigate at the molecular level the precise mechanisms involved in the production of the abnormal traits.

### [Point 8] The relevance of the sampling localities and the possible effects of the tsunami and earthquake

For the collection of the samples, we covered several localities in cities both south and north of the NPP. It is likely that the high contamination levels in the cities northwest of the NPP, including Fukushima, contributed to the *Z. maha* from these localities exhibiting small forewing size and various abnormalities [1]. Although Shiroishi is located further north than Fukushima, we admit that the inclusion of more northern localities might have produced data that were more convincing (see Point 2).

A number of readers were concerned that the adverse effects were a result of the tsunami. All of our sampling localities were far from the coast and were not affected by the tsunami. These localities were of course affected by the earthquake but we do not believe that earthquakes exert harmful effects on this butterfly.

### [Point 9] The relevance of the sample size

Certain commentators argued that our sample size was too small to make definitive conclusions. Indeed, we admit that the number of individuals collected in May was relatively small, 144 adults in total [1]. This is because the first-voltine individuals were not abundant but it was essential to collect the first-voltine population immediately after the accident to evaluate the effects of the radiation on the first generation. Because we used only males to evaluate the forewing-size differences among the collection localities (the females were used for egg collection, which damaged the wings), the number of individuals per locality was relatively small. The number of males used for the forewing-size measurements from Shiroishi, Fukushima, Motomiya, Koriyama, Hirono, Iwaki, Takahagi, Mito, Tsukuba, and Tokyo were 5, 9, 9, 3, 12, 18, 19, 9, 12, and 14, respectively (Supplementary Table 1 in the original paper [1]). Nevertheless, we detected statistically significant forewing differences (see Point 2 and Figure 5). In the series of experiments presented in the original paper [1], we believe that the sample size was ample (5,942 individuals) (Table 1). This number was large enough to examine the statistical significance, which we did in the original paper [1].

**Table 1:** **Number of *Z. maha *individuals used in Hiyama et al. (2012) **[1]

**Field work or laboratory experiment**	**Number of individuals**
Field collection in May 2011 (P_May_)	144
F_1May_ from P_May_	2,516
F_2May_ from F_1May_	538
Field collection in September 2011 (P_September_)	238
F_1September_ from P_September_	1,563
External exposure experiment	401
Internal exposure experiment	542
Total	5,942

We used a relatively small number of field-collected females to produce the F_1_ generation. Therefore, we cannot exclude the possibility that we accidentally collected pathological parents that produced eggs with genes mutated for reasons other than the irradiation from artificial radionuclides. However, we collected female (and male) parents from eight localities in May 2011 and six localities in September 2011; the number of females (and males) used for the egg collection from Shiroishi, Fukushima, Motomiya, Hirono, Iwaki, Takahagi, Mito, Tsukuba, and Kobe were 1(2), 5(3), 2(3), 4(3), 6(3), 4(3), 5(4), 5(3), and 0(0) for May and 0(0), 5(4), 5(2), 3(2), 4(2), 4(2), 0(0), 0(0), and 5(2) for September, respectively. The majority of them produced many progeny exhibiting abnormal traits at levels that were significantly higher than background (see Point 5, Figure 8 and Point 6, Figure 10). Although the number of females used to collect the eggs was relatively small, we believe that this sample size was acceptable.

A number of people claimed that the number of localities we used was insufficient. We partly agree in that higher numbers of localities would yield more accurate results; however, in practice, collecting hundreds of the pale grass blue butterflies within a relatively short time is demanding. In May 2011, four people visited 10 cities in 6 days to collect 144 adults and in September 2011, similarly four people visited 7 cities in 4 days to collect 238 adults. For future studies, we may consider that consistent monitoring in the same set of localities may be as important as increasing the number of localities.

### [Point 10] The relevance of the rearing conditions

The rearing environment is important for the final fitness and phenotype of insects. We used the standard rearing conditions (26 ± 1°C, 16L-8D) under which we have obtained low mortality and low abnormality rates [2]. A small number of the critics disrespectfully claimed that the experimental groups of larvae, especially in the exposure experiments, were not reared correctly, probably because we did not specifically mention rearing conditions for the external exposure experiments. This claim is without basis, and we completely disagree. In all the experiments, the containers were cleaned and supplied with fresh leaves daily or every two days, as were the control groups on the same day. As a matter of course, the rearing conditions for the experimental and control groups were identical.

**Figure 13:**
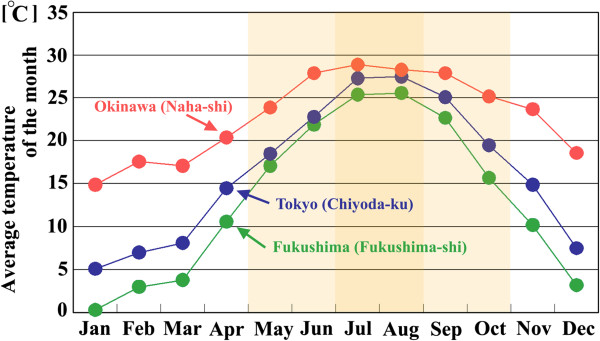
**Average temperatures in Fukushima, Tokyo, and Okinawa from January 2011 to December 2011.** Months with an average temperature of 15°C or more in Tokyo and Fukushima are shaded (May to October). In July and August, the average temperatures in Fukushima were not below 25°C and were similar to Tokyo and Okinawa.

The commentators argued that because *Z. maha* in the Fukushima area is adapted to cold temperatures, rearing the specimens in Okinawa at high temperatures (25-27°C) might have caused harmful effects [66]. We simply disagree. The summer morph in the Fukushima area exhibits relatively high temperatures, and cold temperatures do not occur during this time. According to the Japan Meteorological Agency, Fukushima (Fukushima-shi), Tokyo (Chiyoda-ku), and Okinawa (Naha-shi) exhibit similar temperatures (higher than 25°C) during the summer (July and August) (Figure 13). Months with an average temperature higher than 15°C are found from May to October in Fukushima and Tokyo and from February to December in Okinawa. These periods correspond roughly to the periods of emergence of the *Z. maha* adults in the field.

We have been rearing *Z. maha* from the northern range margins (Fukaura, Aomori Prefecture) and other localities of the Honshu main island but to date, an increase in the death rate or abnormality rate has not been observed in Okinawa. Similarly, we have no evidence of adverse effects arising from feeding the Okinawa individuals the non-contaminated host plants from Honshu or from feeding the Honshu individuals the host plants from Okinawa.

### [Point 11] The importance of the exposure experiments and the possible discrepancy in the radiation sensitivity

**Figure 14:**
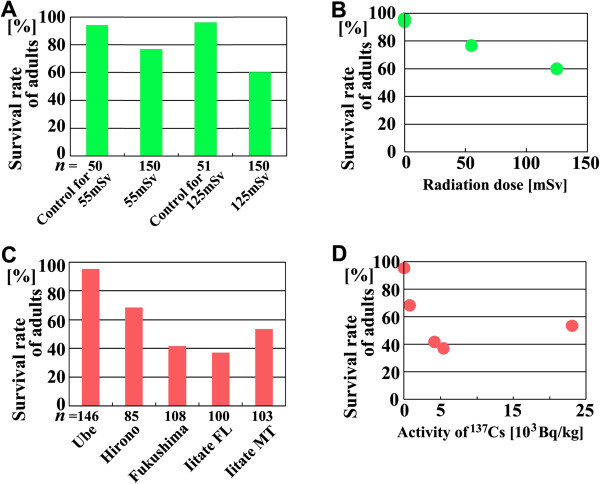
**Survival rates of irradiated individuals in external and internal exposure experiments.** These data were presented in the original paper [1] as a part of the survival curves. **(A)** Survival rates of externally exposed individuals to the adult stage. **(B)** Relationship between the survival rate of the irradiated individuals and the external irradiation dose. At 0 mSv, two plots demonstrate close to 100% survival. **(C)** Survival rates of internally exposed individuals to the adult stage. **(D)** Relationship between the survival rate of adults and the internal irradiation dose.

The most important experiments in the original paper [1] were the exposure experiments, especially the internal exposure experiment (Figure 14). Most opponents did not pay attention to the exposure experiments, despite that (or because) these experiments reproduced the results of the field work and rearing experiments. Because insects, especially Lepidoptera (butterflies and moths), are thought to be highly resistant to radiation exposure [40-42], the dose-dependent decline in the survival rates of adults we obtained [1] may have been unexpected (Figure 14).

An example of a typical argument put forward by the critics was that “these experiments must be nonsense because insects are highly resistant to radiation exposure”. This attitude entirely ignores differences in the experimental conditions. Our experimental conditions (long-term low-dose experiments using *Z. maha*) are entirely new, and reconciliation with the previous data (short-term high-dose experiments) is entirely possible.

To our knowledge, internal exposure experiments of this type (i.e., feeding foods collected from various contaminated localities to animals collected from a non-contaminated locality) have not been performed previously. In this experiment, apart from the simultaneous external exposure in the field, we exactly reproduced what the *Z. maha* in the Fukushima area experience. It is important to emphasise that the conventional short-term high-dose exposure conditions are very different from the long-term low-dose exposure conditions we used in our experiments. Most previous studies in insects used the short-term high-dose radiation exposure of agricultural pests to produce infertile adults [40-42,67-72]. In contrast, in our *Z. maha* experiments, the long-term low-dose exposures were performed continuously from the first- or second-instar larvae to pupae, immediately before eclosion in the external exposure experiment. Therefore, our results cannot be compared with other studies in that respect. At this point, we need to perform confirmatory experiments. We are just beginning to explore the biological effects of long-term low-dose radiation exposure on animals.

In our exposure experiments, the control groups (no exposure) were set and survival curves were compared, yielding statistically significant dose-dependent differences [1] (Figure 14). Both the external exposure (31.7%) and internal exposure (39.6%) demonstrated much higher rates compared with the controls (Figure 11), demonstrating physiological damage in somatic cells. Indeed, in the locality around the NPP, the animals received both external and internal exposures simultaneously, a tougher situation than our experimental settings, which may be why we detected relatively frequent colour-pattern aberrations in the field even in the samples from relatively low-dose regions.

**Figure 15:**
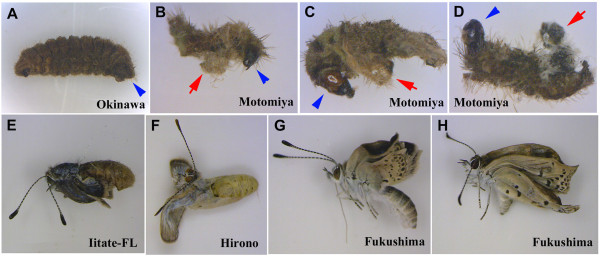
**Ecdysis and eclosion failure in the internal exposure experiment.** The larvae from the Okinawa parents were fed the natural host plant collected from various localities. **(A)** A rare dead larva (dried specimen) that ate leaves from Okinawa (non-contaminated). External morphology of the late instar larva is similar to that of the live one (see Figure 2C, D). **(B-D)** Dead larvae (dried specimens) that ate contaminated leaves from Motomiya. The larvae had partially shed their shells, indicating the failure of ecdysis. Arrows indicate the partially shed shells, and the arrowheads indicate the heads of the larvae. **(E, F)** Dead adults that could not exit from the pupal case. They ate contaminated leaves from the Iitate flatland (FL) or Hirono. **(G, H)** Abnormal adults that failed eclosion despite exiting from the pupal case. They ate contaminated leaves from Fukushima.

Although not quantified, the manner of death of the internally exposed individuals appeared unique; many died because of the failure of ecdysis, pupation, and eclosion (Figure 15). Consistent with our observations, frequent pupal and larval death because of moulting failure and morphological abnormalities in the testes, antennae, and wings induced by irradiation have been documented in other insect systems [73,74].

Ecdysis, pupation, and eclosion are processes that require coordinated cellular activities to shed the external shell. Therefore, cells do not have to be killed directly by the radiation to produce lethality in an individual. The disruption of ecdysis, pupation, and eclosion causes death, for example, when the enzymes for ecdysis [45,75] do not function well. Furthermore, in lepidopteran insects, the DNA is replicated many times in the last-instar larvae, and in the pupal period, the replicated DNAs are distributed to the cells by cell division [45]. Therefore, this period of development is expected to be highly sensitive to radiation exposure.

It has been speculated that environmental stress promotes developmental abnormalities that cause eclosion failure in holometabolous insects [76]. Our results are largely consistent with this speculation. Because stressful environment promotes evolution [77], it is likely that selection against abnormal individuals will be promoted in the Fukushima area.

We here point out that some effects of low-dose exposures have been described in cell culture systems. It has been demonstrated that less than 1 Gy can affect the cell. The biological effects include two types, hyper-radiosensitivity (HRS) and increased radioresistance (IRR) [78-83] in which exposure to less than 0.5 Gy decreases the survival rate considerably (HRS) but further exposure to 0.5-1 Gy increases the resistance (IRR). Although the molecular mechanisms are not apparent, Matsumoto [81] pointed out that HRS occurs because the DNA damage is too minor to activate the DNA repair system, and IRR occurs because the DNA damage is now recognised by the repair system. These low-dose biological effects have been observed in a wide variety of organisms, from protozoa to plants [84-92], including the lepidopteran cell line TN-368 [93].

Additionally, in low-dose exposure, non-targeted effects in non-irradiated cells have been described, which include DNA damage and cell division retardation in the non-irradiated cells (similar to the irradiated cells). The non-targeted effects include the bystander effect (observed following exposure to less than 0.2 Gy in particular) in the non-irradiated cells near the irradiated cells and genomic instability in the F_1_ and F_2_ offspring [27,93-106]. These effects might have occurred in irradiated larvae and pupae in our study.

Yagi (2012) [66] suggested that under the conditions of the external exposure experiment, a *Z. maha* individual might have actually received approximately 1.4 Gy as a combination of β-ray and γ-ray radiations despite that we indicated radiation doses as 55 mSv and 125 mSv for γ-ray (Our measurements were performed using a Hitachi Aloka Medical TCS-161 scintillation survey meter (Tokyo) for measuring up to 30 μSv/h and a RAE Systems DoseRAE2 (San Jose, CA, USA) when measuring more than 30 μSv/h; and a Hitach Aloka Medical TGS-121 GM survey meter). We agree with Yagi’s suggestion because our dose values shown in the paper did not consider β-ray doses. The radiation might have been absorbed on the surface of the larvae and pupae. This interpretation of the damage to epidermal cells is consistent with the difficulties experienced in the ecdysis, pupation, and eclosion that led to death (see above). Yagi (2012) [66] referred to a study demonstrating that *black-striped* mutations occur after the 1.0 Gy irradiation of eggs in *Bombyx mori*[107]. In other words, the doses we used may have been enough to introduce DNA damage or cause other harmful effects.

A number of the commentators argued that the doses we used in the external exposure experiments were higher than the doses experienced in the localities from which we collected our samples, and therefore, the experimental conditions we used did not accurately reproduce the results of the field work; we largely agree. However, it is expected that some of the localities closer to the Fukushima Dai-ichi NPP would have experienced similar doses. The primary purpose of the external exposure experiment was not to quantitatively determine the effects of low-dose levels of irradiation but to qualitatively reproduce the abnormalities, forewing-size reduction, and lethal effects that were obtained in the field-collected samples.

Here, we would like to address three relevant issues. First, at this point, we have not examined whether the abnormal traits that were induced by the external and internal exposures were heritable. Second, in the internal exposure experiment, the highest dose, from the Iitate montane region, did not produce the lowest survival rate (see Figure 13 in this paper and Figure 5d in the original paper [1]). This deviation may be an indication of IRR. Third, a possible alternative explanation for the results of the internal exposure experiment is that the irradiation itself did not result in harmful effects but the irradiated host plant synthesised a stress-induced chemical that killed the larvae. It is true that some plants synthesise defence chemicals following stress; however, this interpretation is difficult for the following reasons. First, the results of the internal exposure experiment were similar to those of the external experiment, which have nothing to do with plant chemicals. Second, plant chemicals usually repel insects; however, if that were the case, the larvae would avoid eating the leaves and would die of hunger, which we did not observe in the internal exposure experiment. The larvae ate the leaves normally and died largely because of the failure of ecdysis, pupation, and eclosion. Third, there is no evidence that the low-dose radiation exposure of plants induces the synthesis of chemicals that are harmful to insects. Fourth, the indirect dose-dependence of the survival rates is more difficult to envision than a direct effect. Importantly, even if this alternative interpretation is correct, the biological effects of the radiation on *Z. maha* are still correct, although effects were indirect. In any case, more studies will be necessary to resolve these issues.

### A possible story of *Z. maha* in the Fukushima area

Frequently, we have been asked about what really occurred in Fukushima; based on our results, what was the story of the *Z. maha* butterfly in the Fukushima area? Synthesising our results, we propose the following.

Following the explosion, the larvae were exposed both externally and internally to radiation, resulting in growth retardation and body-size (wing-size) reduction. In the first-voltine generation (the first generation exposed), both somatic and germ-line cells were damaged; however, the health effects of this damage in this generation were not very severe. In the second-voltine generation and in later generations, the *Z. maha* was fully exposed externally and internally to ionising radiation throughout its entire life stages, resulting in an increase in mortality and abnormalities. Furthermore, mutations in the germ-line cells were passed on to the progeny. Because mutations were introduced continuously because of the continuous irradiation, the mutations accumulated, causing a further increase in the mortality and abnormalities in the later generations. In the adults, the morphological abnormalities, such as the wing colour-pattern aberrations and non-morphological abnormalities, including infertility, became epidemic. At that point, many individuals suffered from genetic diseases in addition to physiological diseases because of the continuous irradiation of the somatic cells.

As a result, the number of individuals that constituted the population decreased. In regions very close to the Fukushima Dai-ichi NPP (e.g., within 10 km of the NPP), it is likely that the *Z. maha* population decreased considerably. Even in the regions from which we sampled, a population-level decline is unavoidable. On the other hand, individuals that are more resistant to irradiation are naturally selected and these individuals survive. Because irradiation directly causes genetic mutations, rare advantageous mutations (e.g., more efficient DNA repair enzymes) will also be produced within a relatively short time. These advantageous genotypes and mutations will penetrate a population relatively quickly, and radiation resistance will evolve in the Fukushima populations. However, we do not know how many generations will be required for this evolution.

## Conclusions

Based on our data, we conclude that the radionuclide contamination from the Fukushima Dai-ichi NPP caused harmful effects on *Z. maha* at physiological and genetic levels. Our results may appear to contradict previous studies of radiation biology; however, the data may not really be contradictory because of differences in the experimental conditions. It is noteworthy that studies of low-dose radiation effects are increasing. However, this study involving both field and laboratory studies that proposes a coherent story using long-term low-dose exposure systems at the organismal level is novel, with the exception of studies of the Chernobyl accident [108]. It is reasonable to suggest that our results are consistent with other field studies performed in Chernobyl and Fukushima [109-111]. Of course, we admit that our data are far from perfect and that further research will be necessary to confirm and clarify several points that were raised by our research [1].

Extrapolating the findings of our and other studies, ecosystems around the Fukushima Dai-ichi NPP face a tremendous challenge. We cannot evaluate the effects on humans directly from the results of the *Z. maha* study. However, considering the exceptionally large amounts of radioactive materials released into the environment, which according to a news release (August 26^th^ 2011) from the Japanese Ministry of Economy, Trade and Industry was 168.5 times as large as the Hiroshima atomic bomb (this is likely a significant underestimate) [28,112,113], underestimating the effects on organisms (including humans) and ecosystems without any experimental evidence is not advisable. We should honestly acknowledge that we are just beginning to understand the effects of long-term low-dose radiation on organisms. Lepidopteran cells are more resistant than human cells to short-term high-dose exposures but in our study, the larvae and pupae were vulnerable to the long-term low-dose exposures at the organismal level. At this point, nobody can scientifically present conclusive data demonstrating that the long-term low-dose exposure in the Fukushima area is safe or unsafe for humans, not to mention for humans with physical or mental weaknesses and those who will be born in the next generation. “No data” should not be confused with “safe”.

## Competing interests

The authors declare that they have no competing interests.

## Authors’ contributions

All authors discussed and approved the content of this paper. JMO directed the project, AH, WT, and JMO prepared the figures, WT performed the statistical analyses and provided the images of the natural behaviour and rearing method, CN provided the images and other data on the internal exposure experiment, MI provided the images and other data on the EMS-induced mutagenesis experiment and the images of the natural behaviour, SK contributed to collecting the data, AH drafted the manuscript, and JMO finalised the manuscript.
